# Genetic variation and population structure of Botswana populations as identified with AmpFLSTR Identifiler short tandem repeat (STR) loci

**DOI:** 10.1038/s41598-017-06365-y

**Published:** 2017-07-28

**Authors:** Tiroyamodimo Tau, Anthony Wally, Thokozile Patricia Fanie, Goitseone Lorato Ngono, Sununguko Wata Mpoloka, Sean Davison, María Eugenia D’Amato

**Affiliations:** 1 University of the Western Cape, Department of Biotechnology, Forensic DNA Laboratory, Private Bag X17, 7535 Bellville, Cape Town South Africa; 2 Botswana Police Service, Forensic Science Laboratory, Private Bag 0400, Gaborone, Botswana; 3 University of Botswana, Biological Sciences Department, Private Bag 00704, Gaborone, Botswana

## Abstract

Population structure was investigated in 990 Botswana individuals according to ethno-linguistics, Bantu and Khoisan, and geography (the nine administrative districts) using the Identifiler autosomal microsatellite markers. Genetic diversity and forensic parameters were calculated for the overall population, and according to ethno-linguistics and geography. The overall combined power of exclusion (CPE) was 0.9999965412 and the combined match probability 6,28 × 10^−19^. CPE was highest for the Khoisan Tuu ethnolinguistic group and the Northeast District at 0.9999582029 and 0.9999922652 respectively. CMP ranged from 6.28 × 10^−19^ (Khoisan Tuu) to 1,02 × 10^−18^ (Northwest district). Using pairwise genetic distances (F_ST_), analysis of molecular variance (AMOVA), factorial correspondence analysis (FCA), and the unsupervised Bayesian clustering method found in STRUCTURE and TESS, ethno-linguistics were found to have a greater influence on population structure than geography. FCA showed clustering between Bantu and Khoisan, and within the Bantu. This Bantu sub-structuring was not seen with STRUCTURE and TESS, which detected clustering only between Bantu and Khoisan. The patterns of population structure revealed highlight the need for regional reference databases that include ethno-linguistic and geographic location information. These markers have important potential for bio-anthropological studies as well as for forensic applications.

## Introduction

Botswana is a landlocked country in Southern Africa. It has 25 languages belonging to the Niger-Congo and Khoisan African language phyla^1^. The Niger-Congo language phylum is widespread through-out sub-Saharan Africa; with languages belonging to its Bantoid branch collectively referred to as Bantu^2^. Botswana Bantu languages are members of the Central-K (Subiya and Mbukushu), Central-R (Yeyi), and Central-S (Kgalagadi, Tswana and Kalanga) Bantu sub-groups^2^. Khoisan languages have distinctive click consonants and are widely used by hunter-gatherer (San) and pastoralist (Khoi) populations of southern Africa^3^. The Khoe-Kwadi, Kx’a, and Tuu Khoisan languages in Botswana are members of the Southern African Khoisan (SAK) family^4–6^. In this study, the terms “Bantu” and “Khoisan” are applied as they relate to linguistics and ethnicity (ethno-linguistics).

Khoisan speakers were the first inhabitants of Botswana^7^. The Khoi pastoralists appear in the archaeological record of southern Africa around 2000–1,200 years ago having migrated from eastern Africa^5, 8, 9^. Bantu speakers originated around the Cameroon/Nigeria border region about 5000–3000 years ago, and only arrived in southern Africa not earlier than the Khoi pastoralists^8^. The Kgalagadi, Tswana and Kalanga of the Central-S Bantu ethno-linguistic sub-group entered present day Botswana between the 15^th^ and the 17^th^ century, the Kalanga from the north (present day Zimbabwe), and the Kgalagadi and Tswana from the south-east (present day South Africa)^7^. The Yeyi (Central-R Bantu ethno-linguistic sub-group) and Mbukushu (Central-K Bantu ethno-linguistic sub-group) moved towards the Okavango Delta (Northwest district) in the 18^th^ century from southwest Zambia, the Caprivi Strip and the Kwando and Linyanti Rivers; while the Subiya (Central-R Bantu ethno-linguistic sub-group) fled into Botswana from the Caprivi Strip in 1875^10^.

There is little information about the genetic and demographic processes that influenced the genetic landscape of Botswana population groups today. In southern Africa, the admixture dynamics between the Bantu farmers and foraging San and pastoralist Khoi played an important role in shaping the current patterns of genetic diversity^11–15^. As the Bantu farmers entered new areas and intermarried with autochthonous populations, the local languages, cultures and genetic composition were influenced or even replaced by the Bantu^16–18^. There is strong evidence of sex-biased gene flow from autochthonous populations to the Bantu^19, 20^, and admixture and assimilation partly involving now extinct autochthonous populations^20^. Besides anthropological evidence, linguistically, click consonants (distinctive to Khoisan languages) have been borrowed into southern African Bantu languages such as the Central-S Bantu Nguni (found in South Africa), and the Central-K Mbukushu and Central-R Yeyi from Botswana^21, 22^.

**Figure 1:**
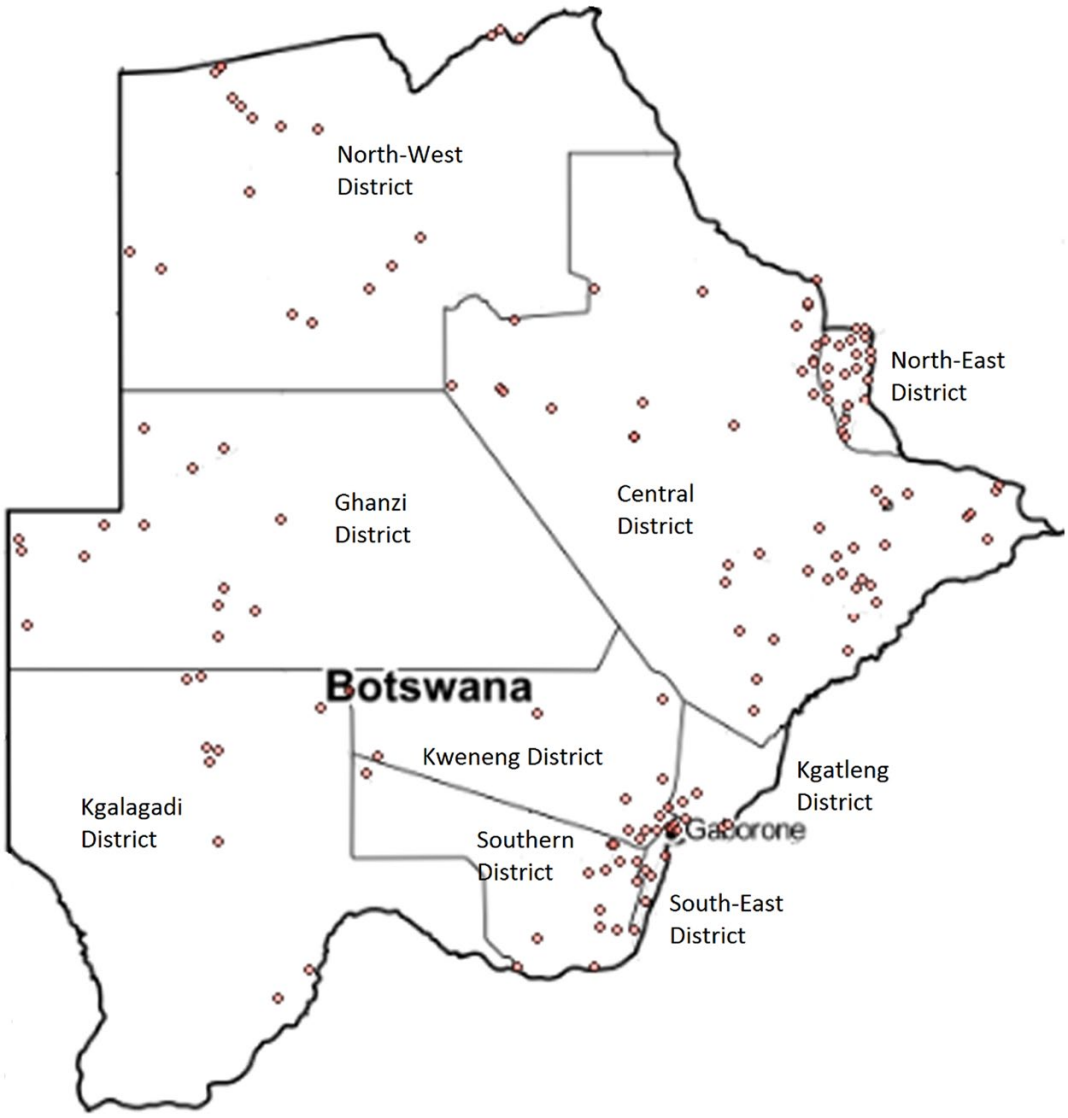
Botswana Administrative Districts and sampling locations made with R 3.2.4^79^ (http://www.R-project.org) package *ggplot2* (https://cran.r-project.org/web/packages/ggmap/citation.html)^80^ and package *ggmap* (https://cran.r-project.org/web/packages/ggplot2/citation.html)^81^.

Today, Botswana has approximately two million inhabitants, of which over 90% are Bantu and only 3% are Khoisan and geographically, the country is divided into nine administrative districts: Central, Ghanzi, Kgalagadi, Kgatleng, Kweneng, Northeast, Northwest, Southeast, and Southern district (see Fig. 1). While the Bantu ethno-linguistic population groups are dispersed throughout the country, the Khoisan ethno-linguistic population groups are mainly concentrated in the sparsely populated central regions of the country near the Kalahari Desert with populations of less than 1 inhabitant per 10 km^2^ (see Supplementary Fig. S1). The majority of the population is concentrated in the south-east regions (Southern and Southeast districts) of the country with the Khoisan groups being concentrated in the Northwest and Ghanzi districts.

The official country census makes no reference to ethnicity because at independence from the British in 1966 the government attempted to create an ethnic-blind state by placing all the Bantu ethno-linguistic groups found in Botswana under the umbrella of the Tswana^23^. Even though no identification of ethnicity is recorded, this information could potentially be inferred from individual genotypic data. Both historic and demographic factors had an influence on the current amount and distribution of genetic variation in Botswana (though the levels of their influence are uncertain)^19, 20^. Evidence of genetic structure between the Bantu and Khoisan has been detected using over 1, 000 nuclear microsatellites in 121 African populations^24^; 1 million autosomal markers in 103 Bantu and Khoisan southern African populations^14^; 900 complete mitochondrial DNA sequences in southern African populations^25^; however this has not been investigated with forensic markers.

Forensic markers have been poorly investigated in Botswana^26, 27^. Results from these few studies indicate high levels of polymorphism detected with the forensic kit AmpFLSTR Profiler Plus PCR Amplification Kit (Applied Biosystems)^26^ (commercial forensic kit preceding AmpFlSTR Identifiler PCR Amplification Kit) and AmpFLSTR Yfiler PCR Amplification Kit Y-STR markers^27^ indicating their potential for forensic and bio-anthropological studies. Analysis with Y-filer markers also revealed a lack of geographic, regional and ethnic variation in Botswana. The Botswana police has adopted the AmpFlSTR Identifiler PCR Amplification Kit (Identifiler) for forensic investigations. Identifiler is a commonly used commercial multiplex PCR kit designed to amplify the 13 core STR loci from the FBI Combined DNA Index System (CODIS), and two additional markers (D2S1338 and D19S433), plus a homologous region of the Amelogenin gene on the X and Y chromosomes. It has been used to generate relevant reference data on various worldwide and African populations^28–43^ as well as elucidating the population structure of samples from Rwanda^28^, Namibia^34^, Sudan^38^, and South Africa^42, 43^.

In this work, the widely utilized forensic kit Identifiler was used to investigate the population structure in Botswana, according to ethno-linguistics and geography. For this, we applied summary statistics, multivariate analysis, and model-based Bayesian clustering methods. These markers were further investigated for their ability to provide ancestry information of random individuals. These analyses of the patterns and distribution of genetic diversity are discussed in a forensic and bio-anthropological context.

## Results and Discussion

### Rare ‘variant’ alleles, ‘off-ladder’ alleles, and tri-allelic patterns

Rare ‘variant’ are alleles that differ from common variants which fall within virtual bins, while ‘off-ladder’ alleles are those that do not size the same as consensus alleles present in the allelic ladder and are not found in the bin set^44^. A total of 15 ‘rare variant’ alleles and 9 ‘off ladder’ alleles were found in this study (Supplementary Table S1). All rare variants have been previously reported (Supplementary Table S1).

Two types of tri-allelic patterns have been distinguished by Clayton *et al*.^45^: Type 1 with two alleles having different peak height intensity to the third allele, and Type 2 with peaks with even intensity. Tri-allelic patterns in this study were only detected at TPOX locus all Type 2 (Supplementary Figure S1). All these involved allele 10, and eight of the 10 samples were female. The extra allele 10 has been theorized as being a translocation of allele 10 onto the X-chromosome (X-linked) as a higher frequency of women have presented with tri-allelic patterns than men^46, 47^. The transmission of the tri-allelic genotype by mothers and fathers showed that fathers only transmitted the tri-allelic pattern to daughters, while the sons and daughters received the tri-allelic genotype equally from their mothers, evidence of X-chromosome inheritance^46^. Lane^46^ hypothesized that the translocation of the extra TPOX allele with the X-chromosome within African populations occurred prior to the Bantu expansions because these occurrences were found in South African, Namibian and Ghanaian populations. Ristow *et al*.^43^, suggested that the driving force of the high frequency of the tri-allelic genotype in South African populations is the cultural practice of polygamy, also practiced in Botswana.

### Genetic diversity parameters

The genetic diversity parameters of the overall Botswana population, and according to ethno-linguistics and geography are shown in Supplementary Table S3 (A-P). Deviations from HWE were found at CSF1PO and D19S433 in the overall population and were due to heterozygote deficiency. The Central-R sub-group from the Bantu ethno-linguistic population groups displayed deviation from HWE at locus D13S317. The Khoisan ethno-linguistic sub-group Tuu showed deviation from HWE at the D19S433 and TPOX locus.

Deviations from HWE were observed for CSF1PO, D19S433, and FGA in the Ghanzi district, and for CSF1PO and D18S51 in the Kgalagadi and Southeast districts respectively. Locus D19S433 is the only locus in which deviations from HWE were found in both ethno-linguistic and geographic population groups. This observation can be linked to the high concentration of Khoisan in the Ghanzi district. Schlebusch *et al*.^39^ also detected departure from HWE for D19S433 in San populations of South Africa. The CSF1PO locus was also seen to deviate from HWE in populations from Uganda (Karamoja)^35^ and Angola^37^.

A review by Dakin and Avise^48^ revealed that silent alleles at frequencies normally reported in literature are unlikely to introduce serious biases in average exclusion probabilities. However, they can introduce errors that may lead to false exclusions of maternity or paternity in individual assessment. Amorim and Carneiro^49^ reported that the presence of silent alleles may lower paternity index (PI) ratios in trios. Therefore, the presence of silent alleles should be taken into account in all forensic analysis. The vWA locus was reported to show high frequencies of silent alleles in South African Bantu and Coloured populations^50^. Therefore, in absence of trio genotypes, we investigated the possibility of silent alleles applying the maximum likelihood method incorporated in ML-NullFreq^51^ (See Supplementary Table S4). No appreciable levels of silent alleles were detected at any locus except for the Khoisan Tuu, with a proportion of 0.17 at locus D19S433. Therefore, the presence of silent allele/s might explain the deviation from HWE and heterozygote deficiency in this ethno-linguistic sub-group.

### Evaluation of population heterogeneity

Summary statistics, *F*_*ST*_ and AMOVA, multivariate methods, and unsupervised model-based Bayesian clustering were used to evaluate population structure in Botswana. The comparative study based on *F*_ST_ showed significant differences between the self-declared Bantu and Khoisan ethno-linguistic groups (n = 990; F_ST_ = 0.01213; *P* = 0.00000). Supplementary Table S5 shows *F*_*ST*_ results comparing the language subgroups of the Bantu (Central-K, -R, -S n = 747) and Khoisan (Khoe-Kwadi, Kx’a, Tuu n = 223). There were significant differences between all the ethno-linguistic subgroups with the exception of Central-K and Central-R Bantus. The *F*_*ST*_ analysis between the administrative districts revealed significant differences between the Ghanzi district and all other districts except the Kgalagadi and Kgatleng districts; between the Southern and Northwest district; and between the Northwest and the Kweneng and Central districts (Supplementary Table S6).

Non-hierarchical AMOVA testing for ethno-linguistic and geographical variation showed greater variation amongst self-declared ethno-linguistic groups than among the geographic groups at 3.30% and 1.54% respectively (Table 1 Test 1 and 3). Hierarchical AMOVA testing for ethno-linguistic variation between the self-declared Bantu and Khoisan ethno-linguistic population groups (n = 970) showed greater within population variation (95.63%) than among populations within groups (1.00%) (Table 1 Test 2). Hierarchical AMOVA testing for geographical variation between the northern and southern districts (n = 990) showed 0.12% variation amongst groups; and higher variation within populations at 98.39% than amongst populations within groups at 1.49% (Table 1 Test 4). Variation among populations within groups was higher when testing for geographic heterogeneity (1.49%) than for ethno-linguistic heterogeneity (1.00%).

**Table 1:** Hierarchical and non-hierarchical analysis of molecular variance (AMOVA) between the different Botswana population groups according to ethno-linguistic (A) and geographic (B) heterogeneity.

Groups		Source of variation	Variation (%)	*F*_*ST*_	*F*_*SC*_	*F*_*CT*_
**A**						
**Ethno-linguistic heterogeneity**						
Test 1 (Non-hierarchical)	(1) Central-K Bantu(2) Bantu: Central-R Bantu(3) Bantu: Central-S Bantu(4) Khoe-Kwadi Khoisan(5) Kx’a Khoisan(6) Tuu Khoisan	Among populations	3.30			
		Within populations	96.70	0.03301*		
Test 2 (Hierarchical)	(1) Bantu: Central-K + Central-R + Central-S (2) Khoisan: Khoe-Kwadi + Kx’a + Tuu	Among groups	3.37			0.03375
		Among populations within groups	1.00		0.01033*	
		Within populations	95.63	0.04373*		
**B**						
**Geographic heterogeneity**						
Test 3 (Non-hierarchical)	(1) Central(2) Ghanzi(3) Kgalagadi(4) Kgatleng(5) Kweneng(6) North-east(7) North-west(8) Southern(9) South-east	Among populations	1.54			
		Within populations	98.46	0.01544*		
Test 4 (Hierarchical)	(1) North: North-west + Ghanzi + Central + North-east (2) South: Kgalagadi + Kweneng + Kgatleng + Southern + South-east	Among groups	0.12			0.00119
		Among populations within groups	1.49		0.01488*	
		Within populations	98.39	0.01606*		

*P < 0.001.

**Figure 2:**
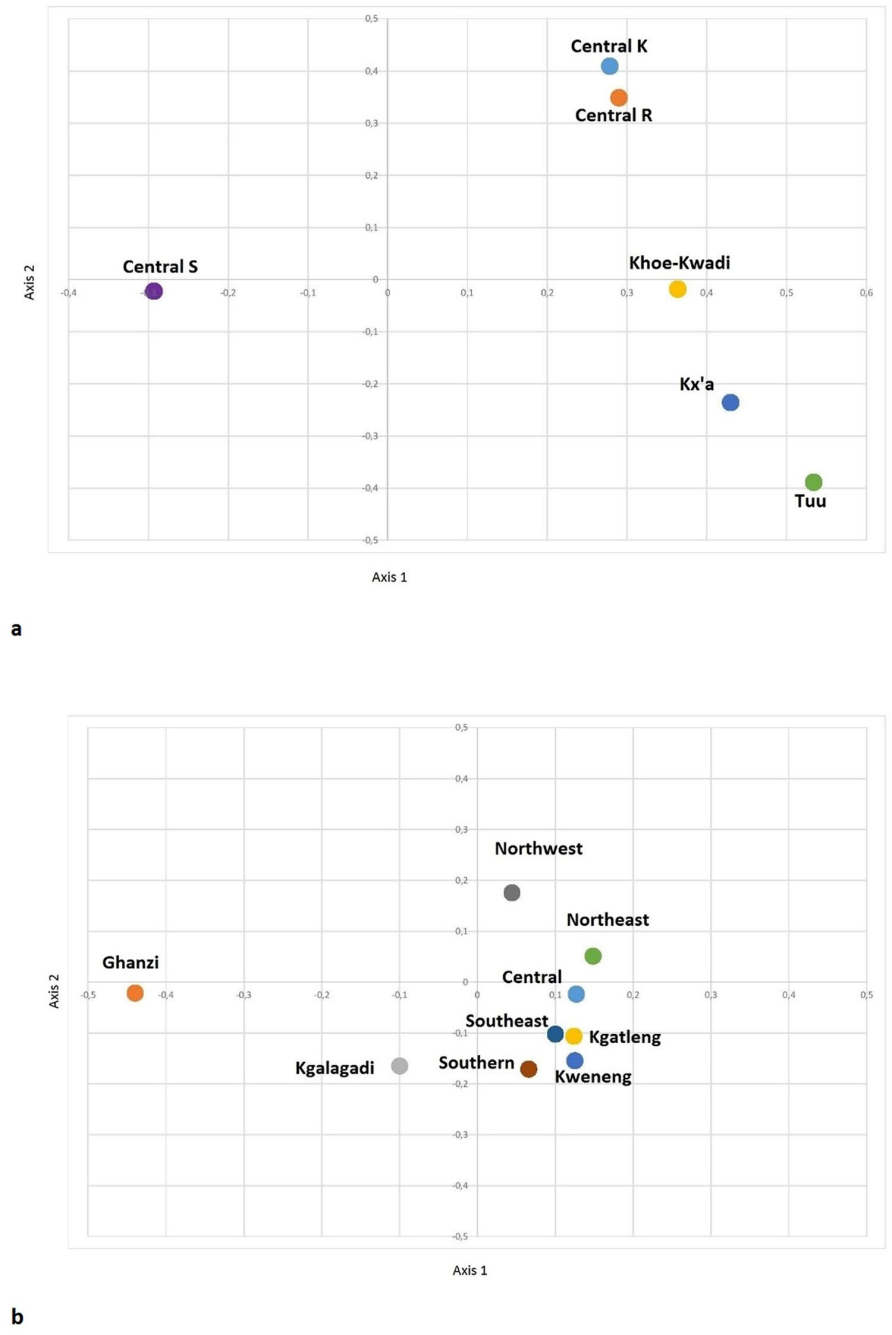
(**a**) Factorial correspondence analysis (FCA) of the language subgroups of the Bantu (Central-K, -R and -S) and Khoisan (Tuu, Kx’a, and Khoe-Kwadi) speaking people of Botswana. (**b)** Factorial correspondence analysis (FCA) of the nine administrative districts of Botswana.

The relationship between Bantu and Khoisan self-declared sub-language ethno-linguistic groups is further illustrated using factorial correspondence analysis (FCA) (Fig. 2a) Bantu Central-S is an outlier. The Bantu Central-K and-R cluster together separated from the Khoisan sub-groups. This is a result of their common origin (southwest Zambia) and subsequent gene flow as they settled in the same geographical region (Northwest district)^10^ The FCA comparing the nine administrative districts illustrated in Fig. 2b shows the Ghanzi district is an outlier. The Northwest, Ghanzi, and Kgalagadi districts are slightly separated from the cluster formed by the remaining six administrative districts. This result illustrates both the close relationship of the Bantu Central-K and -R as well as the districts with Khoisan can be seen as outliers.

*F*_*ST*_, AMOVA, and FCA show evidence of the greater influence of ethno-linguistics on population structure than geography. The partition of genetic variation within Botswana is due to ethno-linguistics which consequently influences the geographic distribution of genetic variation due to the geographic pattern of genetic distribution as evidenced by *F*_*ST*_, AMOVA and FCA data.

**Figure 3:**
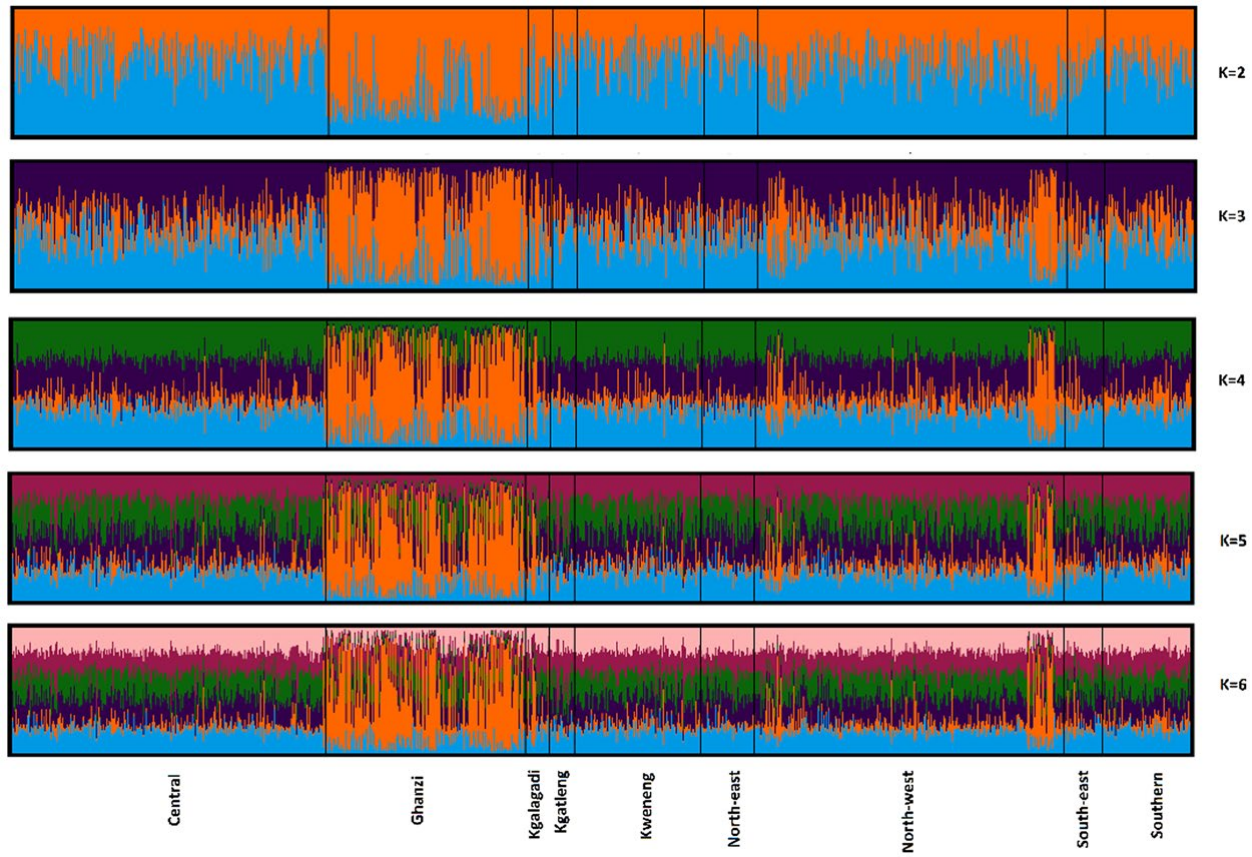
STRUCTURE analysis of Botswana individuals with Identifiler assuming K = 2 to 6. Colours represent the inferred ancestry from K ancestral populations and vertical black lines indicate the nine administrative districts.

**Figure 4:**
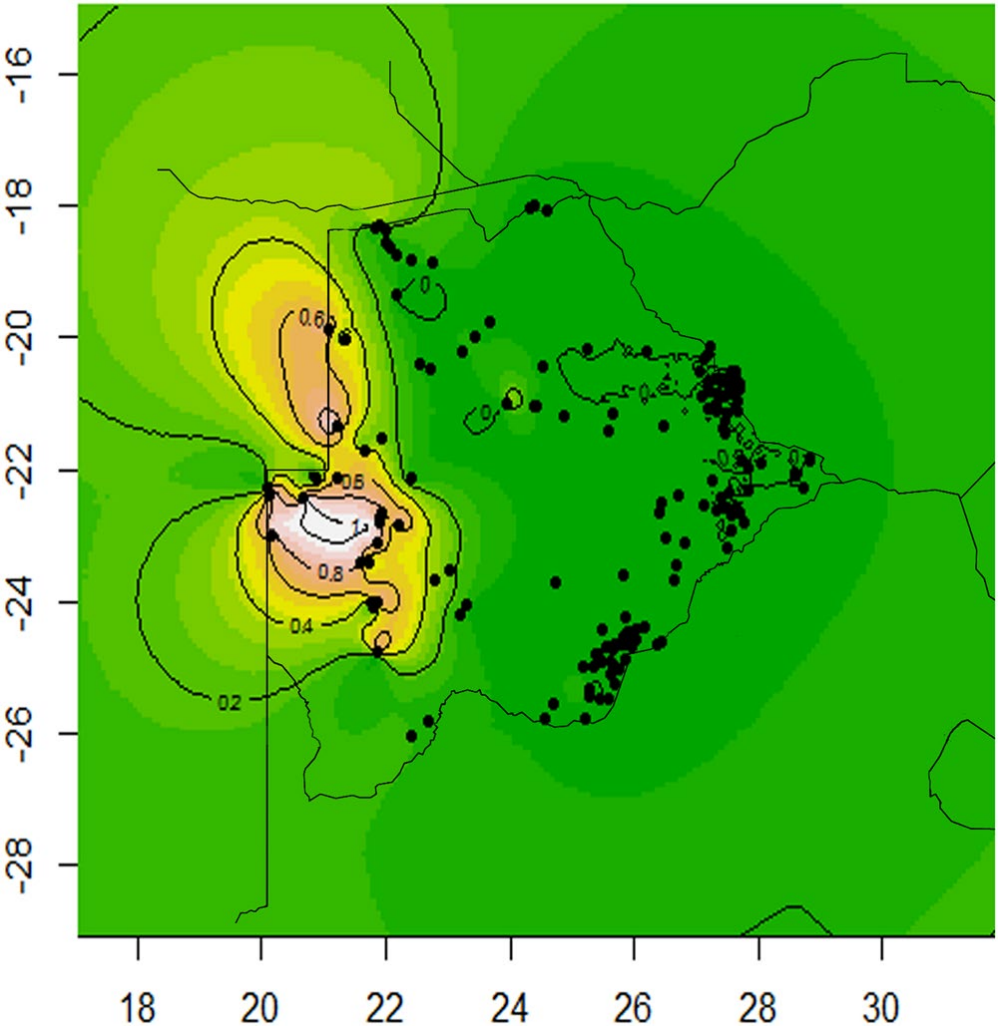
Geographical representation of the admixture coefficients through spatial kriging with low (cool colours) to high (hot colours) representing mean (TESS) admixture proportions. Individuals are classed from the non-admixture analysis in TESS with different colour and/or shapes representing different clusters. The green represents the Bantu and the pink represents the Khoisan. Map created using R 3.0.3^72^ (http://www.R-project.org) package *spatial* 7.3.971^73^ and *maps* 2.3.972^74^.

The results of the model-based cluster analysis method in STRUCTURE are shown in Fig. 3, assuming K = 1–6 ancestral components. The Δ*K* method^52^ identified three populations as having the highest level of genetic structuring. Three genetic clusters were also identified by InP(D | K) as the most likely *K* using the Evanno method^53^. TESS results indicate that there are two district genetic clusters in the Botswana population as seen in the plateau seen in the deviance information criterion (DIC) plot from the no-admixture model implemented in TESS. The ethno-linguistic population groups Bantu and Khoisan are distinguishable at K = 2 up until K = 6. Clustering analysis indicates that the largest concentration of the Khoisan ethno-linguistic group is in the Ghanzi district (Fig. 3). This is in accordance with the geographical data of this study (see Supplementary Table S7). Figure 3 also show two distinct Khoisan clusters in the Northwest district that correspond to two sampling sites on the Okavango Delta (Gudigwa and Xaixai) that are dense in Khoe-Kwadi and Kx’a people respectively. Using TESS results, we were able to show a geographical representation of the admixture coefficients through spatial kriging (Fig. 4). The Bantu and Khoisan ethno-linguistic population groups form distinct clusters with regions of admixture.

STRUCTURE and TESS analysis did not detect substructure within the Bantu ethno-linguistic population groups. However, FCA indicates substructure within the Botswana Bantu ethno-linguistic groups. Even though central and southern African Bantu speaking groups have been found to be genetically similar, the exact patterns of dispersal are still under study. Furthermore, Tishkoff *et al*.^24^ also found that ethno-linguistics and geography explained a significant proportion of the genetic differentiation found in African populations, most of the genetic variation was explained by ethno-linguistics. This is also evident in Botswana, which is consistent with the recent migration of Bantu speakers. The high degree of admixture evidenced in Botswana from the interaction between the Bantu farmers, foraging San, and pastoralist Khoi played an important role in shaping the current patterns of genetic diversity in the country^11–13^. This indicates that these markers have important potential for bio-anthropological studies as well as for forensic applications.

### Assignment and Ancestry

**Figure 5:**
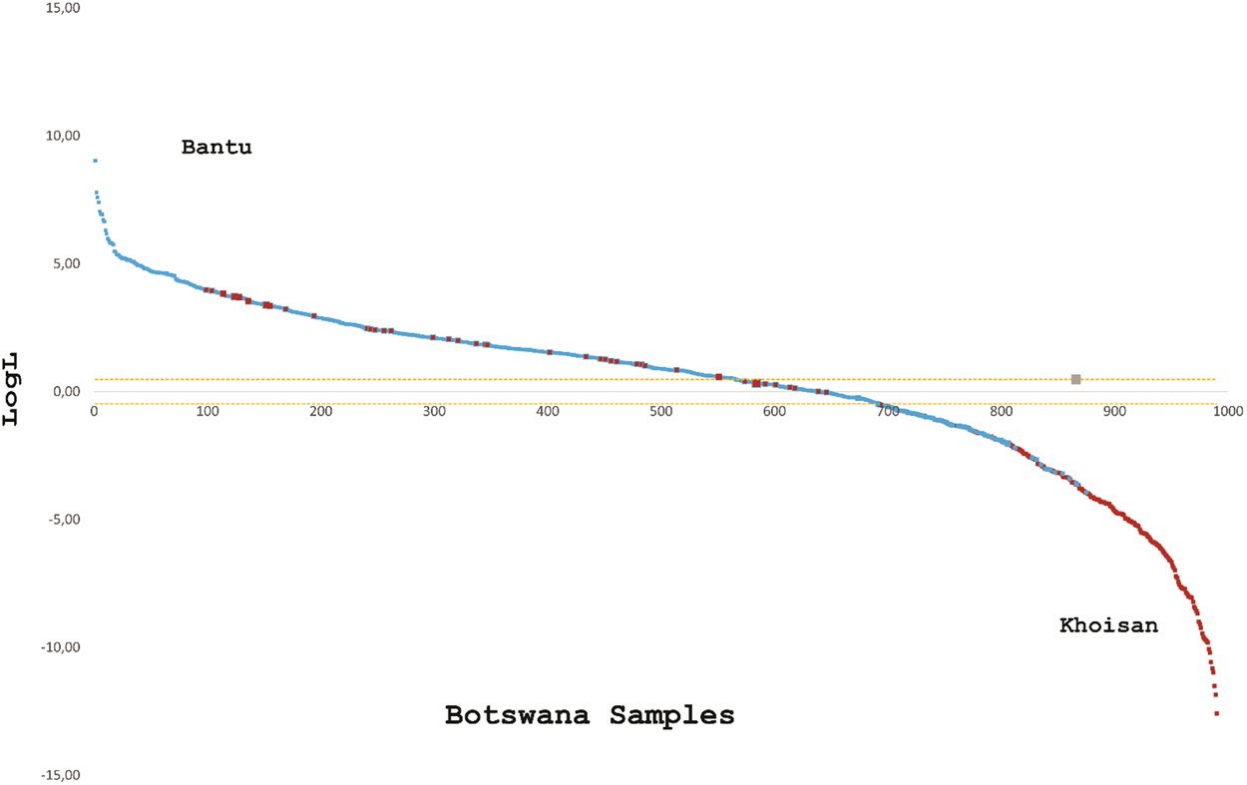
Log likelihood ratios of assignment for all 990 Botswana individuals to Bantu and Khoisan ethno-linguistic groups. The cut off range for Bantu was ≥0.477 (log103) and for Khoisan it was <−0.477. The self-declared Bantu individuals are indicated in blue and the Khoisan in maroon. The samples that fall within the purple dashes represent admixed individuals.

The efficiency of Identifiler to identify ethnic origin (assignment) of the Botswana individuals to the two major ethno-linguistic groups was evaluated using two strategies: log likelihoods using WHICHRUN and proportions of ancestral components from STRUCTURE (Table 2). The ranked log likelihood ratios obtained with each individual are shown in Fig. 5, suggesting an important process of admixture from the Khoisan into the Bantu ethno-linguistic population group. The lowest rate of correct assignment was obtained with STRUCTURE which detected a higher proportion of admixed individuals (597) than WHICHRUN (124). Both methods detected a higher proportion of admixed individuals among self-declared the Bantu (87.7% for STRUCTURE and 91.9% with WHICHRUN) than in the Khoisan (12.1% with STRUCTURE and 8.77% with WHICHRUN). The highest proportions of admixed individuals are from the Central (28.6% using STRUCTURE 29.03% with WHICHRUN) and Northwest (28.8% with STRUCTURE and 34.7% with WHICHRUN) districts.

**Table 2:** Summary results of assignment of individuals to Bantu and Khoisan ethno-linguistic population groups with WHICHRUN (A) and STRUCTURE (B).

Population group	Assigned to				
	Bantu	Khoisan	Not assigned (%)	Correctly assigned (%)	Error rate (%)
**A**					
Bantu	523	111	15.2	69.9	14.8
Khoisan	43	189	4.1	78.1	17.7
**B**					
Bantu	213	28	68.1	28.2	3.7
Khoisan	10	143	35.7	60.1	4.2

The tables show the count of individuals assigned to each ethno-linguistic population group and the proportion of not assigned (admixed), correctly assigned, and wrongly assigned individuals.

The most informative locus for the inference of ancestry was locus CSF1PO (*I*_*n*_ = 0.154), with D7S820 (*I*_*n*_ = 0.036) being the least informative (Table 3). The mean *I*_*n*_ across the markers (and standard deviation) *I*_*n*_ was 0.078 (0.035). This is lower than the average *I*_*n*_ found for the Sudanese populations at 0.167 (0.070)^38^. These low *I*_*n*_ values indicate that individually these markers are not best suited for the inference of ancestry.

**Table 3:** Informativeness of loci for the inference of ancestry *I*_*n*_.

Locus	*I*_*n*_
**CSF1PO**	0.154
**D21S11**	0.130
**D2S1338**	0.111
**D19S433**	0.100
**D18S51**	0.093
**TH01**	0.091
**D5S818**	0.075
**TPOX**	0.073
**FGA**	0.067
**vWA**	0.059
**D3S1358**	0.053
**D16S539**	0.045
**D8S1179**	0.044
**D13S317**	0.044
**D7S820**	0.036
**Average**	0.078
**standard deviation**	0.033

The possibility of assignment of individuals to population groups is beneficial in forensic investigations^54–59^. Autosomal STRs are the markers of choice due to their highly polymorphic nature and significant potential for distinguishing individual identity^32^. These markers have also been used infer the ancestry of profiles^54, 60–62^. The accuracy of assignment depends on marker efficiency and the number of markers. The Identifiler STRs and other forensic markers were not selected for the inference of ancestry but for their ability to distinguish individuals. Algee-Hewitt^63^ found that markers with high individual identifiability also possess high population identifiably and that CODIS loci contain higher ancestry information than randomly chosen STRs.

This study has shown that the Identifiler markers were able to distinguish between the Bantu and Khoisan ethno-linguistic population groups in Botswana. The log-likelihood method is more efficient and faster at assignment than STRUCTURE, resulting in reduced computational time. However, this should be taken carefully due to the important levels of admixture detected in Botswana as a high proportion of individuals who self-declared to belong to the Bantu or Khoisan ethno-linguistic groups were found to be admixed. These results showed the influence of gene flow in the distribution of genetic diversity in Botswana, with a very important incorporation of the Khoisan into the Bantu gene pool seen mostly in the Northwest District. This study has found that the Identifiler markers contain information on population structure supporting findings by Phillips *et al*.^61^, Babiker *et al*.^38^, and Pereira *et al*.^62^.

### Forensic parameters

Forensic parameters for each locus were estimated overall Botswana populations, and according to ethno-linguistics, and geography (Supplementary Table S9). The overall combined power of exclusion (CPE) was 0,9999965412. CPE according to ethno-linguistics ranged from 0,9999582029 (Khoisan Tuu) to 0,999998666 (Khoisan Kx’a); and according to districts ranged from 0,9999922652 (Northeast District) to 0,9999992679 (Kweneng District). Also noticeable is the fact that the Kgalagadi district genotypes did not show any homozygotes and therefore had a CPE value of 1. Compared to other African populations, the CPE was higher for the overall Botswana population than two east African populations (Somalia^36^ and Sudan)^38^ and the South African^40^ populations. It was lower than the southern African Namibia (Ovambo)^34^ and Angola^30^, and the east African Uganda (Buganda^41^ and Karamoja)^35^ populations.

The probability of obtaining a random match between individuals, the combined match probability (CMP), for the overall Botswana population as a whole was 6,28 × 10^−19^. CMP ranged from 6.28 × 10^−19^ (Khoisan Tuu) to 5.91 × 10^−14^ (Bantu Central-K) when tested according to ethno-linguistics; and ranged from 1,02 × 10^−18^ (Northwest district) to 5,12 × 10^−15^ (Kweneng district) when calculated according to geography. The highest CPM was higher than that detected in the South African^39, 40^, Sudanese^38^, and Ugandan (Buganda^41^ and Karamoja)^35^ populations, but lower than that found for the Equatorial Guinea population^29^. Forensic summary statistics results are comparable to other African populations^29–31, 33–41^.

## Conclusions

This study shows the possibility of investigating bio-anthropological processes, admixture, gene flow and major ethnic affiliation of individuals using Identifiler. These markers a suitable in the identification of individuals. This is a step towards closing the gap in understanding the amount and distribution of genetic diversity in Botswana and understand their contributing factors in order to provide recommendations for the application of these markers for the Botswana police. The population structure found in Botswana illustrates the need for regional reference databases that includes ethno-linguistic and geographic location instead of a single national reference database of voluntary donors. This is a much needed step towards creating a regional or national reference DNA database.

## Methods

### Population samples

A total of 990 unrelated voluntary donors (Supplementary Table S7) from the nine administrative districts of Botswana (Fig. 1) were sampled for the study. Of these samples 752 self-declared as Bantu and 238 Khoisan speakers (Supplementary Table S8). Written informed consent was obtained from all the voluntary donors who participated in the study. Blood samples were collected using Whatman FTA cards (Whatman, Maidstone, Kent, UK). DNA was extracted using the Chelex 100 extraction protocol (Bio-Rad) following the manufacturer’s instructions. Approval for this study was provided by the University of Botswana ethics committee and the Ministry of Health Research and Development Committee of Botswana and were carried out in accordance with approved guidelines.

### Genotyping

The samples were amplified using the AmpFlSTR Identifiler (Applied Biosystems, Foster City, CA) kit, containing the loci D8S1179, D21S11, D7S820, CSF1P0, D3S1358, TH01, D13S317, D16S539, D2S1338, D19S433, vWA, TPOX, D18S51, D5S818, FGA, and the Amelogenin locus for sex typing following the user’s manual recommendations. Fragment sizes were detected using the Applied Biosystems ABI 3100 genetic analyser and sized with GeneScan500-LIZ internal size standard (Applied Biosystems, Foster City, CA) following manufacturer’s protocols. Allele calling was performed using GeneMapper ID v 1.1 software (Applied Biosystems, Foster City, CA).

### Analysis of genotype data

#### Genetic Diversity parameters

Genetic diversity parameters such as allele frequency were estimated using Genepop vs 4.2.2^64^. Departures from Hardy-Weinberg equilibrium (HWE), observed (Ho) and expected (He) heterozygosity were calculated using Arlequin vs 3.5.1.3^65^. *P*-values for HWE were executed with 10^6^ steps in the Markov chain and 10^6^ dememorization steps. The Bonferroni adjustment (α = 0.05/15 = 0.00333 for 15 loci) was applied to the probability of HWE to minimize possible type I errors^66^. Heterozygote deficiency or excess as a cause of deviation from HWE was investigated using Genepop. Silent allele frequencies were estimated using a maximum likelihood approach as implemented in ML-NullFreq^51^.

#### Population structure

Population structure was evaluated using different approaches, summary statistics, analysis of molecular variance AMOVA, multivariate methods, and unsupervised model-based Bayesian clustering. Pairwise genetic distances (*F*_*ST*_) and AMOVA were calculated with the program Arlequin vs 3.5.1.3^65^. *F*_*ST*_ *P*-values were estimated at a significance of 0.01 using 10,000 permutations and applied Bonferroni adjustment. AMOVA was run using a F_ST_-like distance matrix at 10,000 permutations. Factorial correspondence analysis (FCA) was conducted using the program GENETIX vs 4.05^67^.

*F*_*ST*_, Hierarchical and non-hierarchical AMOVA, and FCA were used to test for heterogeneity over ethno-linguistic (Bantu Central K, R, and S and Khoisan Khoe-Kwadi, Kx’a and Tuu) and geographic subdivision (administrative districts). Non-hierarchical AMOVA was used to test for ethno-linguistic heterogeneity amongst the self-declared ethnolinguistic groups: (1) Central-K Bantu, (2) Central-R Bantu, (3) Central-S Bantu, (4) Khoe-Kwadi Khoisan, (5) Kx’a Khoisan, (6) Tuu Khoisan (This test was limited to n = 970 because there was no self-declared ethno-linguistic sub-language classifications for twenty donors); and geographic heterogeneity in the nine administrative districts: (1) Central, (2) Ghanzi, (3) Kgalagadi, (4) Kgatleng, (5) Kweneng, (6) Northeast, (7) Northwest, (8) Southern, (9) Southeast (n = 990). Hierarchical AMOVA grouping strategy tested for variation between the self-declared Bantu and Khoisan ethnolinguistic sub-population groups: (1) Bantu- Central-K + Central-R + Central-S; (2) Khoisan- Khoe-Kwadi + Kx’a + Tuu; and geographic variation between the north and south regions of the country: (1) North: Northwest + Ghanzi + Central + Northeast and (2) South: Kgalagadi + Kweneng + Kgatleng + Southern + Southeast.

Population structure was further evaluated using the unsupervised Bayesian clustering methods in STRUCTURE vs 2.3.4^68^ and TESS vs 2.3.1^69^. These two programs were used to take advantage of their different sensitivities to population structure and admixture. STRUCTURE is advantageous in that it is able to explore the number of populations in a dataset by optimizing Hardy-Weinberg equilibrium within putative groups, while as an addition TESS uses geographical information in assigning membership.

STRUCTURE was run using the parameters *admixture* and *correlated allele frequencies model* for a burn-in period of 1,000,000, followed by 100,000 iterations. Ten replicates were run for each K = 1 to K = 6. Summary reports were generated using the STRUCTURE HARVERSTER tool^52^, an ad hoc statistic-based approach which estimates the true number of populations K by estimating ΔK. Individual ancestral components were visualized using CLUMPAK (http://clumpak.tau.ac.il/index.html).

The Bayesian clustering algorithm in TESS was applied using geographical information as an additional parameter in the model. Initially TESS was run with the *no-admixture* model to estimate the upper bound on the number of distinct genetic clusters as recommended in the user manual. The spatial interaction parameter (ψ) was set to 0.6 as recommended^69^. The model was run for 1,000,000 iterations with a burn-in period of 100,000 iterations for K = 2 to K = 10 with ten replicates for each k. The ideal number of clusters (K_max_) was chosen based on when the deviance information criterion (DIC) values reached a plateau (as recommended in the user manual). Using the resulting K_max_, 10 replicates were run using the conditional auto-regressive (CAR) admixture model using the same parameters mentioned above. The average of each individual’s proportions of ancestral components was calculated using the program CLUMPP^70^ over the 10 replicates.

The CAR model uses a hidden regression approach which allows the possibility to display posterior predictive maps of admixture coefficients^71^. These maps provide useful information in addition to the standard unidimensional bar chart representation showing the predictions of admixture proportions for individuals at their geographic locations. The R (http://www.R-project.org) 3.0.3^72^ package *spatial* 7.3.9^73^ and *maps* 2.3.9^74^ were used to map the extent of genetic clusters and identify barriers between clusters using ordinary kriging surface interpolation of admixture proportions. Each of these maps (equal to the number of genetic clusters) extrapolates the admixture proportions (proportion of genotype belonging to that particular cluster) across the study area. Areas with low values in the combined map were considered boundary regions between genetic clusters.

#### Assignment and Ancestry

Using the results of STRUCTURE at K = 2, three groups were identified according to whether their ancestral proportions at a cut-off of 0.7 for ancestral membership proportions for either the Bantu or Khoisan ethno-linguistic groups following criteria similar to Phillips *et al*.^61^ and Ristow *et al*.^43^. Group (1) consisted of individuals who had Bantu ethno-linguistic population group proportions ≥0.7, Group (2) individuals with Khoisan ethno-linguistic population group proportions ≥0.7, and Group (3) individuals whose membership probability proportions were below 0.7 for either the Bantu or Khoisan ethno-linguistic group and therefore admixed. Using proportions of ancestral components, the ability of Identifiler to determine ancestry was evaluated. Those individuals with a K ≥ 0.7 in a group that corresponded to their self-declared ethno-linguistic population group (Bantu or Khoisan) were “correctly assigned”. “Wrongly assigned” meant individuals whose self-declared ethno-linguistic population group showed ancestral proportion ≤0.7. Those whose ancestral components were below 0.7 for both Bantu and Khoisan were considered “admixed”.

The efficiency of assignment of the Identifiler markers was also evaluated using the program WHICHRUN vs 4.1^75^. The genotypes of Group (1)-Bantu and Group (2)-Khoisan were used as a baseline (training set) in WHICHRUN using the critical populations method to determine the log likelihood (log(L)) of the population probabilities for all 990 samples used in the study. The individual genotypes probability ratio limit of 3 (log_10_3 = 0.477) was chosen for population assignment to exclude false positives. An individual was considered “correctly assigned” when assigned to the self-declared population group. An individual was considered “wrongly assigned” when assignment corresponded to other than the self-declared population group. The proportion of wrongly assigned individuals is the error rate. Those individuals that were not assigned to a group were classified as “not assigned” and therefore considered admixed.

The ability of the markers in the inference of ancestry was evaluated in Group (1)-Bantu and Groups (2)-Khoisan using the loci informativeness for assignment (*I*_*n*_) implemented in the program Infocalc^76^. *I*_*n*_ ranges from zero (no information) to the natural logarithm of the number of populations (maximum information). The highest theoretically attainable *I*_*n*_ per locus for this study was 0.693 (log_10_2).

#### Forensic parameters

Standard summary statistics estimating forensic parameters^77^: Power of Exclusion (PE = h^2^(1–2hH^2^) where h and H are the number of heterozygotes and homozygotes respectively, and the Combined $P{E}_{i=1}^{n}$ = 1 − π(1 − PE_i_) were PE_*i*_ is the specific exclusion probability of the ith genetic marker and π(1 − PEi) means (1 − PE_1_) × (1 − PE_2_) × (1 − PE_3_) × … × (1 − PE_n_) from locus *i* = 1 to the *n*th locus, Match Probability (MP = $\sum _{i=a}^{n}\,\sum _{j\ge 1}^{n}{{\rm{P}}}_{{{\rm{ij}}}^{2}}$ where i and j represent the frequencies of all possible alleles a through n. P_ij_ represents the frequencies of all possible genotypes), the combined PM for more than one locus is the product of the individual PM at each locus assuming that they are not linked, Power of Discrimination (PD = 1 − MP), and Typical Paternity Index $({\rm{TPI}}=\frac{H+h}{2H})$ were calculated using an Excel spreadsheet. Polymorphic information content (PIC) was calculated using Cervus vs 3.07^78^.

### Data accessibility

All data used in this study has been deposited into the European Genome-Phenome Archive (EGA) (http://www.ebi.ac.uk/ega/), which is hosted by the European Bioinformatics Institute (EBI), under accession number EGAS00001002380.

## Electronic supplementary material

Supplementary Information
