# Supplementary material for: Genetic variation and population structure of Botswana populations as identified with AmpFLSTR Identifiler short tandem repeat (STR) loci
Source: Sci Rep. 2017 Jul 28;7:6768. doi: 10.1038/s41598-017-06365-y (PMC5533702; doi:10.1038/s41598-017-06365-y)
Supplement: Supplementary file 1 — Supplementary Information [file 41598_2017_6365_MOESM1_ESM.pdf]

Genetic variation and population structure of Botswana populations as identified with AmpFLSTR Identifiler short tandem repeat (STR) loci

Authors: Tiroyamodimo Tau, Anthony Wally, Thokozile Patricia Fanie, Goitseone Lorato Ngono, Senungogo Wata Mpoloka, Sean Davison, María Eugenia D'Amato

Supplementary Information

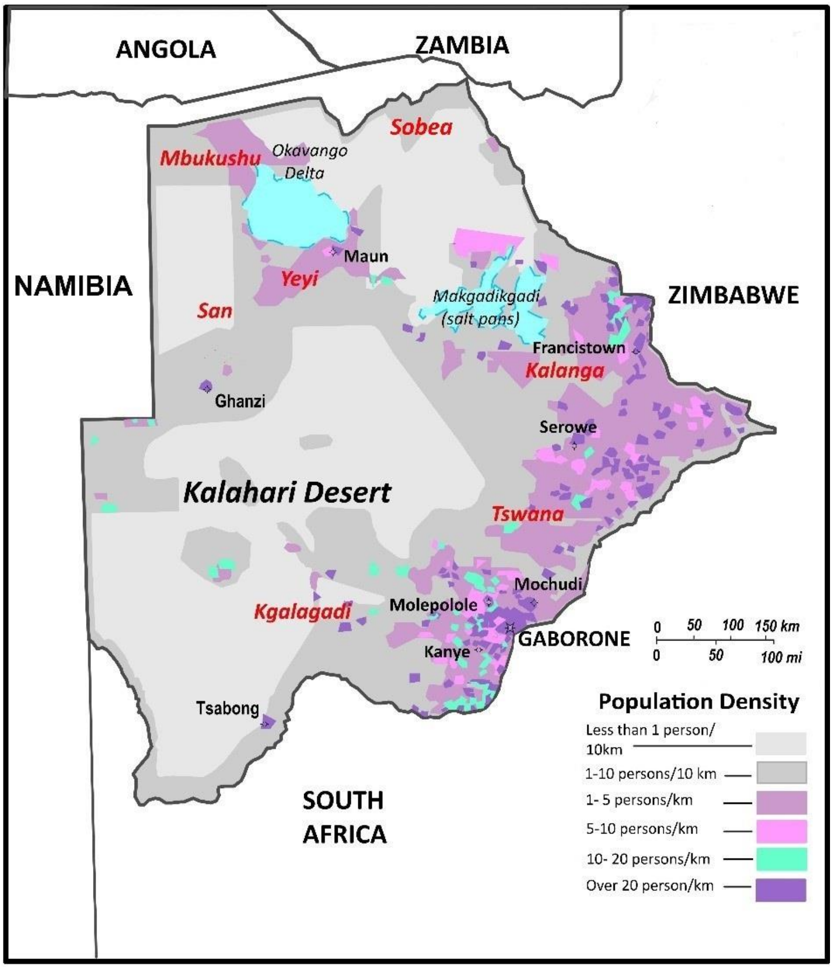

Supplementary Figure S1 Representation of the geographic locations of the different ethno-linguistic language groups used in the study. The Niger-Congo Bantoid branch (Bantu) being: Central S-Bantu-Kgalagadi, Tswana and Kalanga; Central K-Bantu- Yeyi; and Central R-Bantu- Mbukushu. The Khoisan (San) are also seen in the picture. Map adapted from http://www.lib.utexas.edu/maps/africa/botswana-population_density-2005.png (Courtesy of the University of Texas Libraries, The University of Texas at Austin) using Surfer 9 (http://www.ssg-surfer.com).

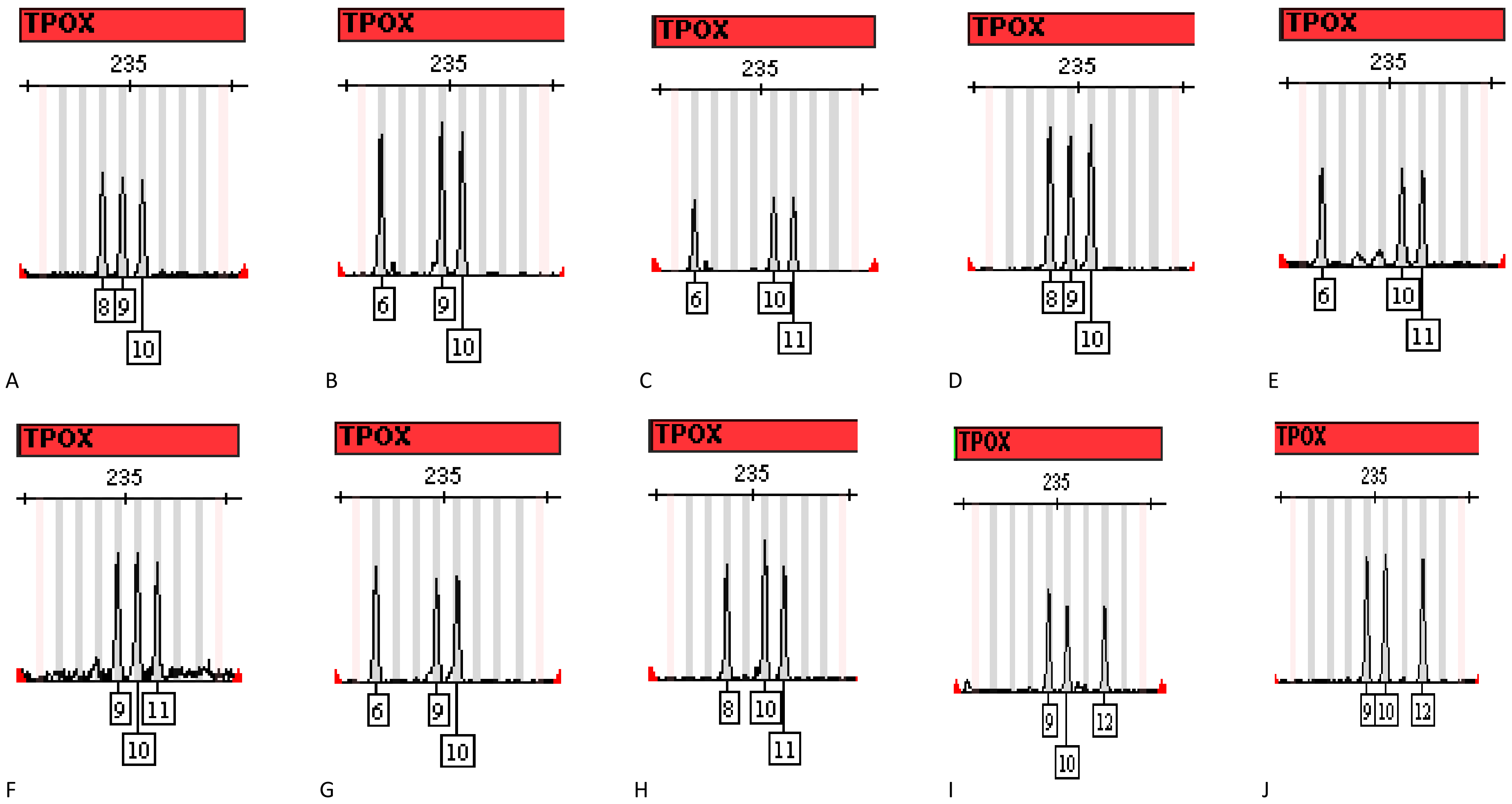

Supplementary Figure S2 Electropherograms for samples exhibiting tri-allelic patters for TPOX. Male samples are D and E.

Supplementary Table S1 Rare “variant” and off-ladder variants observed in Botswana with the Identifiler kit. Off-ladder variants marked with an *. Allele counts in this report are shown along with NIST number of records and publications for African populations along with their reported frequencies in brackets. See Supplementary Table S2 for allele frequencies reported in the study.

**Allele**	**Current Study**							**NIST STR variant database**	**African Published data**		
	**Bantu Central K**	**Bantu Central R**	**Bantu Central S**	**Khoisan Khoe-Kwadi**	**Khoisan Kx'a**	**Khoisan Tuu**	**Undeclared Khoisan**	(http://www.cstl.nist.gov/strbase/var_tab.htm)	**South African**	**Namibia**	**African**
**D21S11 - tetranucleotide repeat ([TCTA] [TCTG])**											
24.3*			3	1				36 reports			
33.1*			10					86 reports	[2]Zulu (0.0102), [6] Bantu (0.0022 – 0.0120), [7] (Bantu 0.0039)		[4] Gabon (0.014), [5] Mozambique (0.004)
34.1*			6	3				21 reports	[1] Coloured (0.0100), [2]Xhosa (0.0042)		
35.1*	1	1	1	3	4			19 reports	[1] Coloured (0.0150), Bantu (0.0100), [2]Zulu (0.0051), Coloured (0.0090)		
36.1*			1					6 reports	[1] Coloured (0.0210)		
37.1*			1					1 report			
39			3		1			1 report	[1] Coloured (0.0050)		
**D7S820 - Tetranucleotide repeat (GATA)**											
5					3			1 report			
**FGA - complex Tetranucleotide repeat ([TTTC]3TTTTTTCT[CTTT]nCTCC[TTCC]2)**											

16			2					8 reports			
18.2		2	8	1				10 reports	[2] Xhosa (0.0042)		[4] Gabon (0.005), [5] Mozambique (0.0292), [8] Mozambique (0.010), [9] Angolan (0.0146)
19.2			9	1				7 reports	[1] Bantu (0.0100), [2] Xhosa (0.0168), Zulu (0.0051)		[3] African (0.0050), [4] Gabon (0.005), [5] Mozambique (0.010), [8] Mozambique (0.0292), [9] Angolan (0.0010)
21.2			8	1				8 reports	[1] Coloured (0.0210), [2] Coloured (0.0045), Xhosa (0.0042), Zulu (0.0051)		
22.2	1		1					19 reports	[1] Coloured (0.0150) [2] Coloured (0.0045), Xhosa (0.0042), Zulu (0.0051)		[3] African (0.0100)
25.2				1				9 reports	[2] San (0.007)		
29.2			1					3 reports	[2] Xhosa (0.0042)		[5] Mozambique (0.003)
**CSF1PO- Tetranucleotide repeat (AGAT)**											
16						1		6 reports			
**D16S539- Tetranucleotide repeat (GATA)**											
17*					1			1 report			
**D19S433- Tetranucleotide repeat (AAGG)(AAAG)(AAGG)(TAGG)[AAGG]n**											
7		4	30	10	5		1	5 reports	[1] Coloured (0.0520), Bantu (0.0500), [2] (Coloured (0.0225)	[1] Khoe (0.0190)	
**D18S51- Tetranucleotide repeat (GAAA)**											
15.2			7	10	2	1		22 reports	[1] Coloured (0.0310), Bantu (0.0100), [2] Xhosa (0.0126), Zulu (0.0051)	[1] Khoe (0.0380)	[3] African (0.0030), [8] Mozambique (0.0033), [9] Angolan (0.0021)
18.2			1					5 reports			[3] African (0.0050)

19.2	1		3					3 reports	[2] Zulu (0.0051)		[3] African (0.0030)
21.1			1					3 reports			
21.2			1					13 reports	[2] Zulu (0.0153)		[9] Angolan (0.0010)

1. Schlebusch, C.M., Soodyall, H. & Jakobsson, M. Genetic variation of 15 autosomal STR loci in various populations from southern Africa. Forensic Science International: Genetics 6, e20-e21 (2012).
2. Ristow, P.G. & Cloete, K.W. GlobalFiler® Express DNA amplification kit in South Africa: Extracting the past from the present. Forensic Science International: Genetics (2016).
3. Phillips, C. et al. Analysis of global variability in 15 established and 5 new European Standard Set (ESS) STRs using the CEPH human genome diversity panel. Forensic Science International: Genetics 5, 155-169 (2011).
4. Steinlechner, M., Schmidt, K., Kraft, H., Utermann, G. & Parson, W. Gabon black population data on the ten short tandem repeat loci D3S1358, VWA, D16S539, D2S1338, D8S1179, D21S11, D18S51, D19S433, TH01 and FGA. International journal of legal medicine 116, 176-178 (2002).
5. Alves, C.n., Gusmão, L., Damasceno, A., Soares, B. & Amorim, A. Contribution for an African autosomic STR database (AmpF/STR Identifiler and Powerplex 16 System) and a report on genotypic variations. Forensic science international 139, 201-205 (2004).
6. Lane, A. et al. Genetic substructure in South African Bantu-speakers: Evidence from autosomal DNA and Y-chromosome studies. American journal of physical anthropology 119, 175-185 (2002).
7. Lucassen, A., Ehlers, K., Grobler, P.J. & Shezi, A.L. Allele frequency data of 15 autosomal STR loci in four major population groups of South Africa. International journal of legal medicine 128, 275-276 (2014).
8. A.C. Semo, Estudo Genético-Populacional dos Principais Grupos de Moçambique-Aplicação Forense, UNIVERSIDADE DA BEIRA INTERIOR, 2013.
9. Melo, M.M. et al. Genetic study of 15 STRs loci of Identifiler system in Angola population. Forensic Science International: Genetics 4, e153-e157 (2010).

Supplementary Table S2 Allele frequencies and Forensic summary statistics for each of the 15 Identifiler STR loci found in the Botswana samples (n=990).

Allele	D8S1179	D21S11	D7S820	CSF1PO	D3S1358	TH01	D13S317	D16S539	D2S1338	D19S433	vWA	TPOX	D18S51	D5S818	FGA
**5**			0.002					0.005							
**6**				0.066		0.077						0.051		0.001	
**7**			0.007	0.053		0.309				0.025		0.026		0.001	
**8**			0.210	0.048		0.386	0.024	0.028				0.311		0.069	
**9**			0.136	0.048		0.169	0.009	0.186		0.002		0.274	0.002	0.035	
**9,3**						0.037									
**10**			0.308	0.233		0.020	0.050	0.146		0.018		0.107	0.011	0.045	
**10,2**													0.006		
**11**	0.027		0.218	0.197		0.002	0.283	0.356		0.057	0.005	0.226	0.001	0.193	
**12**	0.120		0.102	0.291	0.002		0.408	0.170		0.122		0.004	0.037	0.370	
**12,2**										0.034					
**13**	0.192		0.019	0.054			0.157	0.101		0.299	0.010		0.042	0.270	
**13,2**										0.053			0.003		
**14**	0.343			0.008	0.102		0.066	0.007		0.206	0.075		0.040	0.015	
**14,2**										0.067			0.004		
**15**	0.218			0.001	0.335		0.003	0.002	0.018	0.053	0.191		0.122	0.002	
**15,2**										0.031			0.011		
**16**	0.090			0.001	0.330				0.063	0.021	0.264		0.176		0.001
**16,2**										0.012					
**17**	0.009				0.190			0.001	0.068	0.001	0.203		0.164		0.001
**18**	0.001				0.041				0.072	0.001	0.147		0.134		0.010
**18,2**													0.001		0.006
**19**					0.001				0.160		0.077		0.121		0.054
**19,2**													0.003		0.005
**20**									0.088		0.020		0.068		0.057

**21**									0.175		0.005		0.026		0.091
**21,1**													0.001		
**21,2**													0.001		0.005
**22**									0.158		0.002		0.017		0.169
**22,2**															0.002
**23**									0.076		0.001		0.005		0.181
**24**									0.045				0.001		0.185
**24,2**		0.002							0.001						
**24,3**		0.002													
**25**		0.001							0.052				0.004		0.102
**25,2**															0.001
**26**		0.003							0.020				0.002		0.068
**27**		0.064							0.005				0.001		0.019
**28**		0.231							0.001						0.016
**29**		0.132													0.009
**29,2**		0.001													0.001
**30**		0.138													0.002
**30,2**		0.008													0.004
**31**		0.077													
**31,2**		0.072													0.005
**32**		0.020													
**32,2**		0.082													0.002
**33**		0.017													
**33,1**		0.006													
**33,2**		0.034													
**33,3**		0.001													
**34**		0.024													
**34,1**		0.005													
**34,2**		0.008													

**35**		0.034													
**35,1**		0.006													
**35,2**		0.002													
**36**		0.011													
**36,1**		0.001													
**37**		0.012													
**37,1**		0.001													
**38**		0.008													
**39**		0.002													
**42**															0.001
**42,2**															0.007
**43,2**															0.002

Supplementary Table S3 Genetic diversity estimators for the Botswana populations: overall (A), according to the Bantu (B-D) and Khoisan (E-G) ethno-linguistic groups, and according to districts (H-P), The Observed heterozygosity (Hobs), expected heterozygosity (Hexp) and probability of Hardy-Weinberg equilibrium (HWE *P*). Hobs= observed heterozygosity. Significant p values (*P*<0,0033) are in bold. Values out of HWE that show Heterozygote deficiency indicated by an asterisk (*).

	**A**			**B**			**C**			**D**		
	Overall population (n=990)			Bantu Central K (n=43)			Bantu Central R (n=58)			Bantu Central S (n=646)		
**Locus**	**Hobs**	**Hexp**	**HWE *P***	**Hobs**	**Hexp**	**HWE *P***	**Hobs**	**Hexp**	**HWE *P***	**Hobs**	**Hexp**	**HWE *P***
**D8S1179**	0.7727	0.7738	0.0967	0.7441	0.7447	0.2290	0.75862	0.7658	0.9885	0.7879	0.7673	0.0688
**D21S11**	0.8549	0.8854	0.1890	0.8837	0.8738	0.1684	0.81034	0.8574	0.6888	0.8529	0.8717	0.6748
**D7S820**	0.7779	0.7870	0.1144	0.7441	0.7647	0.7829	0.7069	0.7643	0.7171	0.7832	0.7768	0.3912
**CSF1PO**	0.7804	0.8076	**0.0007***	0.7209	0.7865	0.5515	0.7931	0.8115	0.4016	0.7724	0.7959	0.6330
**D3S1358**	0.7201	0.7293	0.8766	0.6976	0.7398	0.2769	0.7069	0.7117	0.7010	0.7275	0.7319	0.4994
**TH01**	0.7111	0.7188	0.2138	0.6976	0.7275	0.2921	0.6724	0.7163	0.1131	0.7182	0.7119	0.1035
**D13S317**	0.7175	0.7264	0.0973	0.5116	0.5641	0.5553	0.5000	0.6699	**0.0014**	0.7414	0.7289	0.6245
**D16S539**	0.7522	0.7718	0.3057	0.7209	0.7326	0.2737	0.77586	0.7823	0.6910	0.7631	0.7616	0.9347
**D2S1338**	0.8575	0.8859	0.1093	0.8604	0.8640	0.6568	0.91379	0.9039	0.7171	0.8498	0.8769	0.2828
**D19S433**	0.8023	0.8399	**0.0006***	0.7907	0.829	0.7670	0.91379	0.8523	0.0062	0.8235	0.8311	0.2338
**vWA**	0.8048	0.8209	0.7287	0.8837	0.8459	0.9779	0.77586	0.8231	0.4979	0.8003	0.8178	0.2902
**TPOX**	0.7509	0.7591	0.1434	0.8139	0.7734	0.3943	0.77586	0.7733	0.1884	0.7631	0.7654	0.9037
**D18S51**	0.8716	0.8831	0.7349	0.8837	0.8730	0.1972	0.7931	0.8729	0.2608	0.8823	0.8761	0.9091
**D5S818**	0.7471	0.7479	0.0076	0.7209	0.7507	0.9813	0.58621	0.6735	0.0253	0.7445	0.7399	0.0800
**FGA**	0.8446	0.8752	0.1581	0.8604	0.8741	0.6342	0.8965	0.8694	0.6888	0.8513	0.8731	0.0695

	E			F			G		
	Khoisan Khoe-kwadi (n=133)			Khoisan Kx'a (n=58)			Khoisan Tuu (n=32)		
**Locus**	**Hobs**	**Hexp**	**HWE *P***	**Hobs**	**Hexp**	**HWE *P***	**Hobs**	**Hexp**	**HWE *P***
**D8S1179**	0.7519	0.7976	0.7088	0.7586	0.8093	0.6632	0.8438	0.7813	0.8805
**D21S11**	0.9023	0.9071	0.4263	0.9483	0.9041	0.2102	0.8438	0.8909	0.0652
**D7S820**	0.7820	0.8135	0.0427	0.8103	0.7939	0.1783	0.7188	0.7589	0.0570
**CSF1PO**	0.8045	0.8075	0.7385	0.7414	0.8000	0.1201	0.7813	0.8036	0.1402
**D3S1358**	0.7143	0.7212	0.7289	0.7241	0.7199	0.2188	0.6563	0.5947	0.8124
**TH01**	0.7368	0.7313	0.6719	0.6897	0.7003	0.2799	0.6875	0.6319	0.5724
**D13S317**	0.6993	0.7271	0.0562	0.7414	0.6891	0.4463	0.6250	0.7574	0.1643
**D16S539**	0.7293	0.8059	0.3992	0.7414	0.7924	0.5114	0.6250	0.8155	0.0584
**D2S1338**	0.8496	0.8861	0.3649	0.9655	0.9039	0.4063	0.9063	0.8958	0.0517
**D19S433**	0.8120	0.8593	0.1875	0.6379	0.7892	0.0609	0.5000	0.8115	**0.0013***
**vWA**	0.8120	0.8206	0.9994	0.6724	0.7712	0.0885	0.6875	0.7753	0.7238
**TPOX**	0.6842	0.7053	0.2157	0.8103	0.7490	0.0490	0.7500	0.6701	**0.0018**
**D18S51**	0.8421	0.8946	0.5419	0.9138	0.8903	0.9802	0.9063	0.9023	0.9808
**D5S818**	0.7444	0.7755	0.0810	0.7241	0.7555	0.0406	0.7813	0.7173	0.6173
**FGA**	0.8196	0.8762	0.1149	0.8966	0.8906	0.0275	0.8750	0.8487	0.1045

	H			I			J			K			L		
	**Central District (n=264)**			Ghanzi District (n=168)			Kgalagadi District (n=20)			Kgatleng District (n=21)			Kweneng District (n=106)		
**Locus**	**Hobs**	**Hexp**	**HWE *P***	**Hobs**	**Hexp**	**HWE *P***	**Hobs**	**Hexp**	**HWE *P***	**Hobs**	**Hexp**	**HWE *P***	**Hobs**	**Hexp**	**HWE *P***
**D8S1179**	0.7917	0.7563	0.1558	0.7738	0.7791	0.8184	0.8500	0.7923	0.9916	0.8095	0.8060	0.4429	0.8208	0.7786	0.2719
**D21S11**	0.8296	0.8677	0.1074	0.8869	0.8984	0.0375	0.8500	0.8885	0.7854	0.8571	0.8966	0.3985	0.8113	0.8683	0.6147
**D7S820**	0.7576	0.7711	0.2422	0.7321	0.8109	0.0318	0.9000	0.8064	0.1632	0.9048	0.7979	0.1585	0.8113	0.7752	0.7307
**CSF1PO**	0.7765	0.7898	0.7569	0.8214	0.8252	0.0023	0.6500	0.8013	0.0076	0.8571	0.8211	0.3982	0.8491	0.8068	0.4976
**D3S1358**	0.7689	0.7403	0.6279	0.6667	0.6784	0.5516	0.7500	0.7397	0.6453	0.7619	0.7549	0.6121	0.7359	0.7089	0.5400
**TH01**	0.7349	0.7014	0.1629	0.7024	0.7183	0.5698	0.6000	0.6180	0.8197	0.7143	0.6771	0.9085	0.6415	0.7206	0.2787
**D13S317**	0.7424	0.7415	0.4299	0.6786	0.6992	0.3339	0.7000	0.7769	0.2006	0.6191	0.6481	0.3524	0.7925	0.7314	0.4064
**D16S539**	0.7538	0.7641	0.4543	0.7202	0.8055	0.0129	0.7500	0.7756	0.7256	0.7143	0.7456	0.2767	0.7642	0.7359	0.3399
**D2S1338**	0.8182	0.8784	0.3588	0.8810	0.8917	0.2036	1.0000	0.8808	0.8813	0.8571	0.8862	0.3985	0.8868	0.8813	0.0073
**D19S433**	0.8220	0.8314	0.6908	0.7262	0.8402	**0.0007***	0.7500	0.8397	0.3050	0.8571	0.9001	0.1621	0.7642	0.8154	0.5287
**vWA**	0.8447	0.8279	0.8909	0.8095	0.8010	0.9790	0.8500	0.8295	0.1221	0.8571	0.8420	0.5947	0.7642	0.8170	0.0466
**TPOX**	0.7424	0.7720	0.7687	0.7202	0.6952	0.0410	0.8000	0.7295	0.3789	0.6191	0.7096	0.1475	0.7736	0.7473	0.1849
**D18S51**	0.8864	0.8748	0.5201	0.8571	0.9000	0.4641	0.8500	0.9051	0.2641	0.9524	0.8432	0.7488	0.8868	0.8774	0.6548
**D5S818**	0.7500	0.7294	0.7622	0.7500	0.7824	0.0486	0.6000	0.7013	0.0730	0.7619	0.7689	0.9127	0.7453	0.7290	0.5017
**FGA**	0.8485	0.8741	0.0710	0.8155	0.8690	0.0018	0.8500	0.8782	0.4449	0.9048	0.9013	0.7433	0.8679	0.8789	0.1010

	M			N			O			P		
	Northeast District (n=45)			Northwest District (n=260)			Southeast District (n=32)			Southern District (n=74)		
**Locus**	**Hobs**	**Hexp**	**HWE *P***	**Hobs**	**Hexp**	**HWE *P***	**Hobs**	**Hexp**	**HWE *P***	**Hobs**	**Hexp**	**HWE *P***
**D8S1179**	0.7111	0.7738	0.5358	0.7462	0.7845	0.2919	0.8784	0.7550	0.2963	0.7973	0.7793	0.2805
**D21S11**	0.8444	0.8652	0.6275	0.9039	0.8958	0.2975	0.9063	0.8720	0.6853	0.8784	0.8590	0.6166
**D7S820**	0.8444	0.7758	0.0172	0.7846	0.7758	0.5823	0.7500	0.7674	0.9807	0.7838	0.7917	0.6176
**CSF1PO**	0.7556	0.7800	0.9794	0.7269	0.7942	0.1754	0.7500	0.8199	0.5721	0.7297	0.8121	0.1355
**D3S1358**	0.6222	0.7239	0.5922	0.7231	0.7358	0.5184	0.6875	0.7282	0.0856	0.6757	0.7411	0.1860
**TH01**	0.7333	0.7086	0.9956	0.7077	0.7258	0.0801	0.6563	0.7406	0.5800	0.8514	0.7289	0.0773
**D13S317**	0.6444	0.7316	0.0586	0.6615	0.6989	0.0142	0.6875	0.7064	0.4648	0.7568	0.7199	0.7431
**D16S539**	0.7111	0.7328	0.9665	0.7731	0.7731	0.1642	0.8125	0.7818	0.4018	0.7162	0.7911	0.2414
**D2S1338**	0.8444	0.8674	0.1625	0.8846	0.8904	0.8162	0.8438	0.8557	0.7053	0.8514	0.8689	0.4514
**D19S433**	0.8444	0.8315	0.5433	0.8346	0.8444	0.5295	0.8750	0.8581	0.5565	0.8108	0.8038	0.2050
**vWA**	0.8667	0.8162	0.9352	0.7269	0.8173	0.0619	0.8438	0.7986	0.9537	0.7162	0.8014	0.1353
**TPOX**	0.7333	0.7718	0.7762	0.7885	0.7768	0.0456	0.6563	0.7634	0.6332	0.7973	0.7570	0.7061
**D18S51**	0.8000	0.8582	0.2877	0.8654	0.8841	0.7004	0.8438	0.8765	0.0024	0.9324	0.8865	0.4182
**D5S818**	0.7556	0.7636	0.3835	0.7077	0.7258	0.3855	0.7813	0.7361	0.4285	0.6622	0.7605	0.1607
**FGA**	0.7556	0.8527	0.1670	0.8692	0.8751	0.4001	0.7813	0.8447	0.6009	0.9324	0.8800	0.4232

Supplementary Table S4 Maximum Likelihood (ML) Silent allele frequencies for the Botswana populations: overall (A). according to the Bantu (B-D) and Khoisan (E-G) ethno-linguistic groups. and according to districts (H-P).

	A	B	C	D	E	F	G
	Overall population (n=990)	Bantu Central K (n=43)	Bantu Central R (n=58)	Bantu Central S (n=646)	Khoisan Khoe-kwadi (n=133)	Khoisan Kx'a (n=58)	Khoisan Tuu (n=32)
**Locus**							
**D8S1179**	0.006	0	0	0.002	0.027	0.017	0
**D21S11**	0.012	0	0.031	0.012	0	0	0.025
**D7S820**	0.006	0	0.023	0	0.009	0	0.044
**CSF1PO**	0.014	0.022	0	0.005	0	0.024	0.031
**D3S1358**	0.002	0.023	0	0	0	0.015	0
**TH01**	0	0.02	0.013	0	0	0	0
**D13S317**	0.01	0.008	0.083	0	0.022	0	0.083
**D16S539**	0.009	0	0	0	0.032	0.012	0.09
**D2S1338**	0.013	0	0	0.017	0.017	0	0
**D19S433**	0.024	0.027	0	0.006	0.034	0.078	0.17
**vWA**	0.01	0	0.019	0.001	0	0.052	0.038
**TPOX**	0.005	0	0	0	0.015	0	0.009
**D18S51**	0.009	0.013	0.045	0	0.018	0	0
**D5S818**	0.013	0	0.031	0.004	0.018	0.023	0
**FGA**	0.008	0	0	0.01	0.019	0	0.004

	H	I	J	K	L	M	N	O	P
**Locus**	**Central District (n=264)**	**Ghanzi District (n=168)**	**Kgalagadi District (n=20)**	**Kgatleng District (n=21)**	**Kweneng District (n=106)**	**Northeast District (n=45)**	**Northwest District (n=260)**	**Southeast District (n=32)**	**Southern District (74)**
**D8S1179**	0	0.001	0	0	0	0.041	0.021	0.02	0
**D21S11**	0.023	0.004	0	0	0.037	0	0.001	0	0
**D7S820**	0.004	0.036	0	0	0	0.012	0	0	0
**CSF1PO**	0.007	0.011	0.051	0	0	0	0.019	0.01	0.009
**D3S1358**	0	0	0	0	0	0.042	0.009	0.026	0.032
**TH01**	0	0.013	0	0	0.02	0	0	0.014	0
**D13S317**	0	0.016	0.018	0	0	0.039	0.024	0	0
**D16S539**	0	0.041	0.017	0.02	0	0.009	0	0	0.044
**D2S1338**	0.037	0.003	0	0.021	0.007	0.004	0	0	0
**D19S433**	0.012	0.07	0.032	0	0.017	0	0.005	0	0
**vWA**	0	0	0	0.006	0.024	0	0.04	0	0.045
**TPOX**	0.012	0	0	0.063	0	0.014	0	0.038	0
**D18S51**	0	0.013	0.036	0	0.003	0.022	0.015	0.004	0
**D5S818**	0	0.024	0.023	0	0	0	0.014	0	0.048
**FGA**	0.01	0.018	0.02	0	0.003	0.037	0	0	0

Supplementary Table S5 Pairwise genetic distance (F_{ST}) (below diagonal) and P-values (above diagonal) of the different Bantu and Khoisan ethno-linguistic groups in Botswana. Significant values ($P<0.003$) are indicated by an asterisk *.

	Central K	Central R	Central S	Khoe	Kx'a	Tuu
**Central K**	-	0.21226	0.00158	0.00000	0.00000	0.00000
**Central R**	0.00174	-	0.00020	0.00000	0.00000	0.00000
**Central S**	0.00402*	0.00377*	-	0.00000	0.00000	0.00000
**Khoe**	0.01363*	0.00921*	0.01024*	-	0.00000	0.00000
**Kx'a**	0.02312*	0.01905*	0.02026*	0.00656*	-	0.00000
**Tuu**	0.03069*	0.02819*	0.03009*	0.01622*	0.02183*	-

Supplementary Table S6 Pairwise genetic distance (F_{ST}) (below diagonal) and P-values (above diagonal) of the nine administrative districts in Botswana. Significant values ($P<0.0003$) are indicated by an asterisk *.

	Central	Ghanzi	Kgalagadi	Kgatleng	Kweneng	Northeast	Northwest	Southeast	Southern
**Central**	-	0.00000	0.19444	0.78071	0.11118	0.88457	0.00000	0.18384	0.09395
**Ghanzi**	0.01456*	-	0.02792	0.00050	0.00000	0.00000	0.00000	0.00000	0.00000
**Kgalagadi**	0.00208	0.00571	-	0.75775	0.12771	0.23681	0.35393	0.44897	0.30344
**Kgatleng**	-0.00171	0.01017	-0.00298	-	0.65855	0.78052	0.50045	0.43996	0.57519
**Kweneng**	0.00071	0.01792*	0.00288	-0.00128	-	0.23235	0.00000	0.10593	0.06881
**Northeast**	-0.00125	0.01623*	0.00276	-0.00217	0.00101	-	0.50718	0.68290	0.07663
**Northwest**	0.00269*	0.01238*	0.00107	0.00011	0.00589*	0.00005	-	0.02505	0.00000
**Southeast**	0.00141	0.01190*	0.00058	0.00044	0.00231	-0.00091	0.00349	-	0.50431
**Southern**	0.00104	0.01226*	0.00144	-0.00061	0.00161	0.00254	0.00526*	-0.00004	-

Supplementary Table S7 Forensic statistics metric for each locus for the Botswana populations: the overall population (A), according to the Bantu (B-D) and Khoisan (E-G) ethno-linguistic groups, and according to districts (H-P). Discrimination capacity (DC), match probability (MP), combined random match probability, polymorphic information capacity (PIC), typical paternity index (TPI), combined paternity index, power of exclusion (PE), and combined power of exclusion (CPE).

A

	**Overall population (n=990)**				
**Locus**	PE	MP	PD	PIC	TPI
**D8S1179**	0.558	0.086	0.914	0.742	2.2
**D21S11**	0.585	0.053	0.947	0.805	2.4
**D7S820**	0.558	0.079	0.921	0.752	2.2
**CSF1PO**	0.549	0.061	0.939	0.782	2.2
**D3S1358**	0.459	0.119	0.881	0.684	1.7
**TH01**	0.452	0.124	0.876	0.673	1.7
**D13S317**	0.439	0.119	0.881	0.678	1.7
**D16S539**	0.511	0.079	0.921	0.747	2.0
**D2S1338**	0.72	0.024	0.976	0.876	3.6
**D19S433**	0.492	0.112	0.888	0.70	1.9
**vWA**	0.582	0.056	0.944	0.794	2.3
**TPOX**	0.516	0.097	0.903	0.723	2.0
**D18S51**	0.744	0.025	0.975	0.873	3.9
**D5S818**	0.477	0.107	0.893	0.705	1.8
**FGA**	0.698	0.029	0.971	0.862	3.3
**Combined**	0.999996541	6.28×10^{-19}			

	B					C					D				
	Bantu Central K (n=43)					Bantu Central R (n=58)					Bantu Central S (n=646)				
**Locus**	**PE**	**MP**	**PD**	**PIC**	**TPI**	**PE**	**MP**	**PD**	**PIC**	**TPI**	**PE**	**MP**	**PD**	**PIC**	**TPI**
**D8S1179**	0.499	0.131	0.868	0.700	1.9	0.524	0.091	0.909	0.724	2.0	0.585	0.0934	0.906	0.732	2.4
**D21S11**	0.662	0.104	0.895	0.849	3.0	0.520	0.074	0.925	0.834	2.0	0.607	0.0568	0.943	0.7925	2.5
**D7S820**	0.163	0.287	0.712	0.718	0.9	0.240	0.226	0.773	0.718	1.1	0.387	0.2179	0.782	0.742	1.5
**CSF1PO**	0.336	0.142	0.857	0.745	1.3	0.391	0.136	0.863	0.779	1.5	0.382	0.1405	0.859	0.767	1.5
**D3S1358**	0.424	0.127	0.872	0.685	1.6	0.439	0.143	0.856	0.651	1.7	0.477	0.1215	0.878	0.685	1.8
**TH01**	0.424	0.137	0.862	0.671	1.6	0.421	0.143	0.856	0.656	2.2	0.457	0.1335	0.866	0.661	1.7
**D13S317**	0.197	0.254	0.745	0.493	1.0	0.179	0.177	0.822	0.608	0.9	0.468	0.1295	0.870	0.685	1.8
**D16S539**	0.336	0.214	0.785	0.683	1.3	0.450	0.172	0.827	0.740	1.7	0.404	0.1525	0.847	0.729	1.5
**D2S1338**	0.715	0.051	0.948	0.839	3.5	0.823	0.033	0.966	0.887	5.8	0.696	0.0283	0.971	0.864	3.3
**D19S433**	0.496	0.094	0.905	0.799	1.9	0.651	0.210	0.789	0.830	2.9	0.485	0.1116	0.888	0.697	1.8
**vWA**	0.7623	0.056	0.943	0.815	4.3	0.555	0.066	0.933	0.791	2.2	0.607	0.0584	0.941	0.793	2.5
**TPOX**	0.599	0.680	0.320	0.730	2.5	0.240	0.308	0.691	0.728	1.1	0.126	0.3990	0.601	0.726	0.8
**D18S51**	0.795	0.071	0.928	0.849	5.0	0.585	0.045	0.954	0.852	2.4	0.747	0.0312	0.968	0.863	4.0
**D5S818**	0.363	0.162	0.837	0.704	1.4	0.196	0.225	0.774	0.610	1.0	0.399	0.1568	0.843	0.697	1.5
**FGA**	0.702	0.050	0.949	0.849	3.4	0.772	0.053	0.946	0.848	4.5	0.680	0.0346	0.965	0.860	3.1
**Combined**	0.999991	5.91×10^{-14}				0.999976	1.05×10^{-14}				0.999986	6.28×10^{-16}			

	E					F					G				
	Khoisan Khoe-kwadi (n=133)					Khoisan Kx'a (n=58)					Khoisan Tuu (n=32)				
**Locus**	**PE**	**MP**	**PD**	**PIC**	**TPI**	**PE**	**MP**	**PD**	**PIC**	**TPI**	**PE**	**MP**	**PD**	**PIC**	**TPI**
**D8S1179**	0.5130	0.074	0.925	0.764	2.0	0.524	0.071	0.928	0.774	2.0	0.682	0.103	0.896	0.733	3.2
**D21S11**	0.6531	0.041	0.925	0.896	2.9	0.898	0.08	0.920	0.887	10	0.487	0.113	0.886	0.866	1.9
**D7S820**	0.4557	0.200	0.799	0.783	1.7	0.543	0.242	0.757	0.756	2.1	0.378	0.283	0.716	0.708	1.5
**CSF1PO**	0.3294	0.182	0.817	0.782	1.3	0.209	0.160	0.839	0.763	1.05	0.330	0.157	0.842	0.764	1.3
**D3S1358**	0.2409	0.308	0.691	0.675	1.1	0.466	0.152	0.847	0.667	1.8	0.363	0.212	0.787	0.552	1.4
**TH01**	0.4875	0.118	0.881	0.687	1.9	0.412	0.145	0.854	0.655	1.6	0.409	0.197	0.802	0.581	1.6
**D13S317**	0.4174	0.133	0.866	0.686	1.6	0.418	0.197	0.802	0.651	1.6	0.226	0.173	0.826	0.703	1.0
**D16S539**	0.3662	0.124	0.875	0.775	1.4	0.409	0.156	0.843	0.754	1.6	0.165	0.163	0.836	0.775	0.9
**D2S1338**	0.6941	0.030	0.969	0.871	3.3	0.930	0.037	0.962	0.887	14.5	0.808	0.064	0.935	0.870	5.3
**D19S433**	0.5214	0.103	0.896	0.843	2.0	0.232	0.142	0.857	0.763	1.1	0.202	0.094	0.905	0.769	1.0
**vWA**	0.6216	0.057	0.942	0.793	2.6	0.386	0.101	0.898	0.728	1.5	0.409	0.099	0.900	0.725	1.6
**TPOX**	0.4507	0.591	0.408	0.653	1.7	0.187	0.500	0.500	0.707	1	0	0.555	0.444	0.611	0.5
**D18S51**	0.6490	0.027	0.972	0.882	2.8	0.817	0.036	0.963	0.871	5.6	0.795	0.04	0.960	0.880	5
**D5S818**	0.4202	0.156	0.843	0.742	1.6	0.407	0.158	0.841	0.708	1.5	0.477	0.210	0.789	0.658	1.8
**FGA**	0.6051	0.038	0.961	0.861	2.5	0.754	0.062	0.937	0.872	4.2	0.736	0.0884	0.911	0.815	3.8
**Combined**	0.999978	$1.21x10^{-15}$				0.999999	$1.52x10^{-14}$				0.999958	$6.28x10^{-19}$			

	H					I					J				
	Central District (n=264)					Ghanzi District (n=168)					Kgalagadi District (n=20)				
**Locus**	**PE**	**MP**	**PD**	**PIC**	**TPI**	**PE**	**MP**	**PD**	**PIC**	**TPI**	**PE**	**MP**	**PD**	**PIC**	**TPI**
**D8S1179**	0.584	0.103	0.897	0.719	2.4	0.551	0.086	0.914	0.742	2.2	0.695	0.105	0.895	0.737	3.3
**D21S11**	0.609	0.066	0.934	0.784	2.5	0.769	0.028	0.972	0.887	4.4	0.695	0.060	0.940	0.853	3.3
**D7S820**	0.523	0.088	0.912	0.735	2.1	0.480	0.066	0.934	0.781	1.9	0.795	0.140	0.860	0.752	5.0
**CSF1PO**	0.556	0.076	0.924	0.758	2.2	0.639	0.062	0.938	0.800	2.8	0.355	0.135	0.865	0.751	1.4
**D3S1358**	0.543	0.120	0.880	0.695	2.2	0.379	0.156	0.844	0.632	1.5	0.510	0.140	0.860	0.673	2.0
**TH01**	0.484	0.151	0.849	0.646	1.9	0.432	0.120	0.880	0.677	1.7	0.291	0.179	0.821	0.557	1.3
**D13S317**	0.497	0.108	0.892	0.699	1.9	0.396	0.138	0.862	0.656	1.6	0.428	0.130	0.870	0.720	1.7
**D16S539**	0.906	0.090	0.910	0.730	1.3	0.460	0.069	0.931	0.774	1.8	0.510	0.110	0.890	0.725	2.0
**D2S1338**	0.633	0.028	0.972	0.865	2.8	0.757	0.028	0.972	0.879	4.2	1.000	0.075	0.925	0.845	0
**D19S433**	0.640	0.050	0.950	0.811	2.8	0.470	0.049	0.951	0.820	1.8	0.510	0.085	0.915	0.798	2.0
**vWA**	0.684	0.056	0.944	0.803	3.2	0.617	0.071	0.929	0.770	2.6	0.695	0.125	0.875	0.782	3.3
**TPOX**	0.497	0.089	0.911	0.733	1.9	0.460	0.166	0.834	0.643	1.8	0.599	0.170	0.830	0.671	2.5
**D18S51**	0.768	0.033	0.967	0.860	4.4	0.709	0.022	0.978	0.889	3.5	0.695	0.065	0.935	0.872	3.3
**D5S818**	0.510	0.124	0.876	0.684	2.	0.510	0.088	0.912	0.748	2.	0.291	0.185	0.815	0.625	1.3
**FGA**	0.692	0.032	0.968	0.860	3.3	0.628	0.039	0.961	0.853	2.7	0.695	0.075	0.925	0.840	3.3
**Combined**	0.999999563	7.27×10^{-18}				0.99999684	2.14×10^{-18}				1.000000	5.21×10^{-15}			

	K					L					M				
	Kgatleng District (n=21)					Kweneng District (n=106)					Northeast District (n=45)				
**Locus**	**PE**	**MP**	**PD**	**PIC**	**TPI**	**PE**	**MP**	**PD**	**PIC**	**TPI**	**PE**	**MP**	**PD**	**PIC**	**TPI**
**D8S1179**	0.617	0.116	0.884	0.754	2.6	0.638	0.098	0.902	0.740	2.8	0.446	0.094	0.906	0.730	1.7
**D21S11**	0.709	0.061	0.939	0.864	3.5	0.620	0.035	0.965	0.851	2.7	0.684	0.049	0.951	0.842	3.2
**D7S820**	0.805	0.152	0.848	0.746	5.3	0.620	0.094	0.906	0.737	2.7	0.684	0.136	0.864	0.730	3.2
**CSF1PO**	0.777	0.135	0.865	0.775	3.3	0.693	0.081	0.919	0.776	3.3	0.519	0.083	0.917	0.738	2.0
**D3S1358**	0.530	0.143	0.857	0.691	2.1	0.486	0.147	0.853	0.654	1.9	0.318	0.123	0.877	0.665	1.3
**TH01**	0.451	0.179	0.821	0.601	1.8	0.344	0.121	0.879	0.669	1.4	0.482	0.144	0.856	0.647	1.9
**D13S317**	0.314	0.202	0.798	0.580	1.3	0.585	0.126	0.874	0.684	2.4	0.348	0.128	0.872	0.681	1.4
**D16S539**	0.451	0.138	0.862	0.697	1.8	0.534	0.107	0.893	0.701	2.1	0.446	0.119	0.881	0.688	1.7
**D2S1338**	0.709	0.075	0.925	0.851	3.5	0.769	0.040	0.960	0.865	4.4	0.684	0.056	0.944	0.842	3.2
**D19S433**	0.709	0.070	0.930	0.868	3.5	0.534	0.059	0.941	0.790	2.1	0.684	0.064	0.936	0.802	3.2
**vWA**	0.709	0.088	0.912	0.798	3.5	0.534	0.073	0.927	0.789	2.1	0.728	0.075	0.925	0.780	3.8
**TPOX**	0.314	0.170	0.830	0.645	1.3	0.551	0.124	0.876	0.700	2.2	0.096	0.095	0.905	0.725	4.4
**D18S51**	0.903	0.093	0.907	0.800	10.5	0.769	0.037	0.963	0.860	4.4	0.599	0.053	0.947	0.832	2.5
**D5S818**	0.530	0.111	0.889	0.714	2.1	0.502	0.131	0.869	0.680	2.0	0.519	0.105	0.895	0.716	2.0
**FGA**	0.805	0.061	0.939	0.868	5.3	0.730	0.037	0.963	0.862	3.8	0.519	0.057	0.943	0.826	2.0
**Combined**	0.999999929	$5.12x10^{-15}$				0.99999927	$5.12x10^{-15}$				0.99999227	$1.14x10^{-16}$			

	N					O					P				
	Northwest District (n=260)					Southeast District (n=32)					Southern District (74)				
Locus	PE	MP	PD	PIC	TPI	PE	MP	PD	PIC	TPI	PE	MP	PD	PIC	TPI
D8S1179	0.503	0.079	0.921	0.752	2.0	0.565	0.129	0.871	0.705	2.3	0.594	0.098	0.902	0.740	2.5
D21S11	0.803	0.024	0.976	0.885	5.2	0.808	0.057	0.943	0.844	5.3	0.751	0.042	0.958	0.839	4.1
D7S820	0.571	0.086	0.914	0.739	2.3	0.510	0.098	0.902	0.716	2.	0.569	0.084	0.916	0.753	2.3
CSF1PO	0.471	0.068	0.932	0.763	1.8	0.510	0.074	0.926	0.782	2.0	0.476	0.068	0.932	0.782	1.9
D3S1358	0.465	0.119	0.881	0.688	1.8	0.409	0.162	0.838	0.669	1.6	0.392	0.123	0.877	0.690	1.5
TH01	0.440	0.122	0.878	0.678	1.7	0.364	0.117	0.883	0.688	1.5	0.698	0.165	0.835	0.677	3.4
D13S317	0.371	0.136	0.864	0.653	1.5	0.409	0.154	0.846	0.654	1.6	0.521	0.140	0.860	0.664	2.1
D16S539	0.550	0.088	0.912	0.739	2.2	0.622	0.119	0.881	0.734	2.7	0.454	0.088	0.912	0.753	1.8
D2S1338	0.764	0.024	0.976	0.879	4.3	0.683	0.059	0.941	0.825	3.2	0.698	0.044	0.956	0.849	3.4
D19S433	0.665	0.043	0.957	0.827	3.0	0.745	0.066	0.934	0.827	4.0	0.510	0.112	0.888	0.707	2.0
vWA	0.471	0.057	0.943	0.791	1.8	0.683	0.096	0.904	0.756	3.2	0.454	0.078	0.922	0.767	1.8
TPOX	0.578	0.091	0.909	0.740	2.4	0.364	0.102	0.898	0.709	1.5	0.594	0.112	0.888	0.711	2.5
D18S51	0.725	0.026	0.974	0.871	3.7	0.683	0.086	0.914	0.848	3.2	0.862	0.040	0.960	0.869	7.4
D5S818	0.440	0.119	0.881	0.683	1.7	0.565	0.150	0.850	0.675	2.3	0.372	0.098	0.902	0.716	1.5
FGA	0.733	0.032	0.968	0.861	3.8	0.565	0.061	0.939	0.811	2.3	0.862	0.043	0.957	0.861	7.4
Combined	0.99999863	$1.02x10^{-18}$				0.99999835	$5.56x10^{-16}$				0.999999666	$4.25x10^{-17}$			

Supplementary Table S8 Population samples according to district and self-declared ethno-linguistic population group used in the study (n=990).

District	n	Khoisan	Bantu
**Central**	264	9	255
**Ghanzi**	168	156	12
**Kgalagadi**	20	6	14
**Kgatleng**	21	-	21
**Kweneng**	106	1	105
**Northeast**	45	-	45
**Northwest**	260	66	194
**Southeast**	32	-	32
**Southern**	74	-	74
**total**	990	238	752

Supplementary Table S9 Population samples according to their self-declared Bantu and Khoisan ethno-linguistic population groups (n=990).

Language Family	Branch	Sub-group	n
**Niger-Congo**	Narrow Bantu (Bantu)	Central K[1]	43
		Central R[2]	58
		Central S[3]	646
		Undeclared Bantu	5
**Khoisan**	Southern African Khoisan (SAK)	Khoe-Kwadi	133
		Kx'a	58
		Tuu	32
		Undeclared Khoisan	15

[1]Central K- Subiya, Mbukushu

[2]Central R- Yeyi

[3]Central S- Kgalagadi, Kalanga, Tswana (and its dialects-Barolong, Bakwena, Bangwaketse, Balete, Bakgatla, Barolong, Bangwato, and Batawana)
